# M. Georgini on Remedying the Insalubrity of Marshes

**Published:** 1826-07

**Authors:** 


### ON THE INSALUBRITY OF THE AIR OF MARSHES IN COMMUNICATION WITH THE SEA. BY M. GEORGINI, OF LUCCA.*

The deleterious influence of marshes on the health of those who live in their neighbourhood, is but too well known, and is a subject which well merits the attention both of the physician and the legislator. It is also well known, though hitherto not clearly accounted for, that all marshes, even when close together, and consequently under the same climatorial circumstances, are not equally inimical to human health and human life. Of this fact Italy offers a striking example. In certain parts of that interesting country, the vicinity of marshes does not diminish the fertility or the population;—while, in other localities, it exercises the most baneful powers. It was long ago conjectured, but not proved, that admixture of sea-water with that of marshes, increased the malignity of the exhalations issuing thence, and the problem would now appear to be solved by events which have taken place in Italy.

Between the Ligurian Apennines and the Mediterranean sea, lies a marshy tract of coast, about 12 Italian miles in length, and varying from two to four in breadth, traversed by several mountain streams or rather torrents, which are discharged into the ocean, or into the morasses bordering thereon. The marshy plain in question may be considered as an alluvion deposited by the rivers Arno and Serchio, and is bounded on the sea-line by a sort of embankment, only a few feet above the level of the ocean. The waters collected by rains, &c. are discharged from three basins into the sea by natural or artificial canals. The level of the stagnant waters is below high-water mark, and somewhat above the ocean during ebb tide. In consequence of this circumstance, and before

* Archives Générales.

any hydraulic works were constructed, the flood-tide changed the currents of the different exutories, and caused them to run backwards into the morasses, mixed, of course, with a proportion of sea water. While this was the state of things, the population of this wretched district was very scanty; and Viareggio, now a large town, consisted of only a few huts. The natives, who were few in number, were constant victims to diseases of the liver and spleen—the children were sickly—and old men were no where to be seen. The unhealthiness of the place had, in fact, arisen to such a height, that the culture of the olive tree, with which this fertile tract abounded, was almost entirely abandoned to strangers, who, of course, fell annual victims to the malaria of the marshes. Various attempts were made to remedy the evil; and at length, about the year 1741, a complete stop was put to it, by the construction of valvular gates, which permitted the efflux of the waters from the marshes, but prevented any reflux of water from the ocean. The effect was instantaneous and surprising. The insalubrity disappeared immediately these flood-gates were completed, and only partially re-appeared when they were out of order, and permitted the admixture of salt and fresh water in the marshes. Viareggio is now so salubrious as to be much frequented by the neighbouring wealthy inhabitants, as a place for sea-bathing and enjoying the delightful sea-breezes in the heat of summer. The population has rapidly increased, as a matter of course, since the happy change in the climate, and Viareggio, which, in 1733, contained only 330 inhabitants, now shews a population of between four and five thousand.

That this fortunate change was owing to the means above-mentioned, we are not disposed to deny; nor do we doubt that the admixture of salt and marshy water may have a deleterious influence in the production of malaria; but it is also unquestionable that the most deleterious exhalations issue from morasses which have no communication whatever with the sea.

---

